# Low-dose steroids are associated with indeterminate QuantiFERON-TB Gold In-Tube assay results in immunocompetent children

**DOI:** 10.1038/s41598-021-86053-0

**Published:** 2021-03-19

**Authors:** Kyu Ho Kim, Ji-Man Kang, Jong Gyun Ahn

**Affiliations:** 1 Department of Pediatrics, Severance Children’s Hospital, Yonsei University College of Medicine, 50-1 Yonsei-ro, Seodaemun-gu, Seoul, 03722 Korea; 2 Institute for Immunology and Immunological Diseases, Yonsei University College of Medicine, Seoul, Korea

**Keywords:** Immunology, Microbiology, Biomarkers

## Abstract

Immunocompromised status can result in indeterminate QuantiFERON-TB Gold In-Tube (QFT-GIT) results, but the association of indeterminate results with immunocompetent status in children is unknown. Therefore, we aimed to identify factors associated with indeterminate QFT-GIT results for immunocompetent children. We conducted a retrospective chart review of children (aged ≤ 18 years) who underwent QFT-GIT between September 2006 and July 2017 at the Severance Hospital, Seoul, South Korea. Of the 2037 QFT-GIT assays included in the present study, 7.7% yielded indeterminate QFT-GIT results. Multivariable logistic regression analysis identified younger age (OR 0.88; 95% CI 0.836–0.927; *P* < 0.001), elevated white blood cell (WBC) count (OR 1.066; 95% CI 1.020–1.115; *P* = 0.005), decreased albumin levels (OR 0.505; 95% CI 0.316–0.807; *P* = 0.004), and low-dose steroid therapy (< 1 mg/kg per day of prednisone or equivalent for < 2 weeks) (OR 76.146; 95% CI 8.940–648.569; *P* < 0.001) as significant factors influencing indeterminate results. Younger age, high WBC count, low albumin levels, and low-dose steroid therapy were associated with indeterminate QFT-GIT results. Low-dose steroid therapy had the highest OR for the indeterminate results compared to other significant risk factors. Our study suggests that screening for steroid doses is important prior to performing interferon-gamma release assays for immunocompetent children.

## Introduction

Tuberculosis (TB) poses a serious threat to public health worldwide as it is the leading cause of death from a single infectious disease agent; its mortality rate exceeds that of malaria and HIV/AIDS^1^. Globally, 10 million incident cases of TB occurred in 2018, these included approximately 1 million (11%) TB cases in children under the age of 15. However, the mortality rate was higher in children younger than 15 years old, accounting for 14% of the total deaths. This is higher than the mortality rate for all incident cases, suggesting poorer diagnosis and treatment for children. Additionally, children, especially those under the age of two, are more likely to progress from latent TB infection (LTBI) to active TB than adults^2^. Therefore, early identification and treatment of LTBI in children is an important priority for effectively controlling TB.

Diagnostic tools for LTBI in children include the tuberculin skin test (TST) and interferon-gamma release assay (IGRA). Of late, IGRAs are increasingly being used and supported by national guidelines to diagnose TB^3, 4^; however, their use in children is still limited. There are two major IGRAs available, namely, the QuantiFERON-TB (QFT) Gold assay (Cellestis/Qiagen, Carnegie, Australia) and the T-SPOT-TB assay (Oxford Immunotec Ltd., Abingdon, United Kingdom). IGRAs measure the levels of interferon-gamma (IFN-γ) produced via sensitized T cells in response to stimulation with *Mycobacterium tuberculosis*-specific antigens, including early-secretory antigenic target-6 (ESAT-6) and 10 kDa culture filtrate protein (CFP-10)^5^. Therefore, IGRAs are not affected by the BCG vaccination status or most nontuberculous mycobacterial infections^6^. The QFT Gold In-Tube (QFT-GIT) assay is an enzyme-linked immunosorbent assay (ELISA)-based whole blood test that includes TB7.7 as an additional *M. tuberculosis*-specific antigen. QFT-GIT results are reported as either determinate (positive or negative) or indeterminate. Indeterminate results can either occur when there is a high response to the negative control or a low response to the positive control. In the clinic, indeterminate QFT-GIT results make it difficult for clinicians to diagnose TB. Immunosuppressed children and adults are at risk of obtaining indeterminate QFT-GIT results^7–9^. However, even immunocompetent individuals may receive indeterminate QFT-GIT results. Additionally, the risk of indeterminate QFT-GIT results for immunocompetent children is currently unknown.

Therefore, the aim of the present study was to identify the factors associated with indeterminate QFT-GIT assay results in immunocompetent children.

## Results

### Baseline characteristics

**Figure 1:**
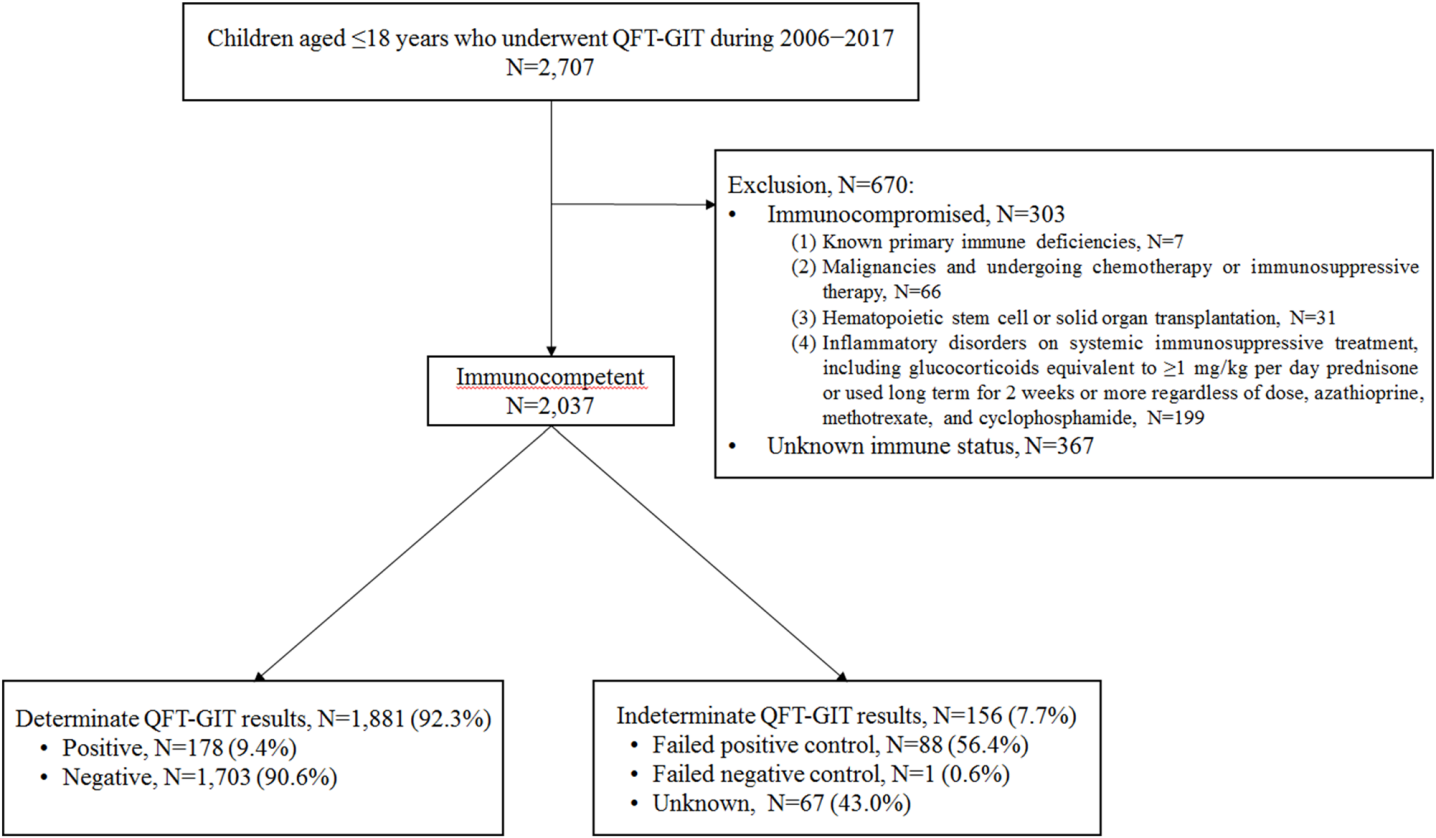
Flow chart presenting the protocol for inclusion in the present study. *QFT-GIT* QuantiFERON-TB Gold In-Tube.

During the study period, a total of 2707 QFT-GIT assays were performed. A total of 670 samples were excluded based on the medical chart review. The exclusion criteria are presented in Fig. 1. Therefore, 2037 QFT-GIT assays were included in the present study. Overall, 1881 (92.3%) tests yielded determinate results and 156 (7.7%) produced indeterminate results. Of the 1881 determinate results, 178 (9.4%) were positive and 1703 (90.6%) were negative. Regarding the 156 indeterminate results, 88 (56.4%) were due to positive control failure, 1 (0.6%) due to negative control failure, and 67 (43.0%) had an unknown cause due to the absence of detailed results.

### Factors associated with indeterminate QFT-GIT results

Comparisons made between the indeterminate and determinate groups are summarized in Table 1. Univariable logistic regression analysis revealed the number of individuals with suspected active TB and unknown reason for IGRA test, white blood cell (WBC) count, C-reactive protein (CRP), erythrocyte sedimentation rate (ESR), and low-dose systemic steroid use was higher in the indeterminate group than that in the determinate group. However, the age at sampling, number of individuals screened for LTBI after exposure to TB patient(s), and albumin levels were lower in the indeterminate group than in the determinate group. Moreover, multivariable logistic regression analysis revealed that age at sampling (OR 0.88; 95% CI 0.836–0.927; *P* < 0.001), WBC count (OR 1.066; 95% CI 1.020–1.115; *P* = 0.005), albumin level (OR 0.505; 95% CI 0.316–0.807; *P* = 0.004), and low-dose systemic steroid use (OR 76.146; 95% CI 8.940–648.569; *P* < 0.001) were significantly associated with the attainment of indeterminate QFT-GIT results.

**Table 1:** Risk of attainment of indeterminate QFT-GIT results in immunocompetent children.

Patient characteristics	Indeterminate (N = 156)	Determinate (N = 1881)	Univariable analysis		Multivariable analysis	
	Median (IQR) or N (%)	Median (IQR) or N (%)	OR (95% Cl)	*P* value	OR (95% Cl)	*P* value
Median age at sampling in years (IQR)	5 (2–11)	8 (3–14)	0.951 (0.924–0.979)	< 0.001	0.880 (0.836–0.927)	< 0.001
Male (N [%])	92 (59.0%)	1020 (54.2%)	0.824 (0.591–1.148)	0.252		
**Reason for IGRA test (N [%])**						
Suspected active TB	101 (64.7%)	860 (45.7%)	2.180 (1.551–3.065)	< 0.001	0.618 (0.330–1.157)	0.133
LTBI screening after exposure to TB patient(s)	14 (9.0%)	683 (36.3%)	0.173 (0.099–0.302)	< 0.001	0.835 (0.097–7.199)	0.870
LTBI screening before starting biologics	11 (7.1%)	141 (7.5%)	0.936 (0.495–1.769)	0.839		
Unknown	30 (19.2%)	197 (10.5%)	2.035 (1.331–3.112)	0.001		
**Laboratory findings; median (IQR)**						
WBC (10^3^/μL)	10,075 (7260–15,030)	7720 (6120–10,040)	1.127 (1.090–1.165)	< 0.001	1.066 (1.020–1.115)	0.005
Lymphocytes (10^3^/μL)	1963 (1110–2932)	2065 (1447–2965)	0.940 (0.834–1.059)	0.308		
Albumin (g/dL)	3.6 (3.2–4.1)	4.2 (3.8–4.5)	0.313 (0.237–0.414)	< 0.001	0.505 (0.316–0.807)	0.004
CRP (mg/dL)	12.6 (2.2–57.0)	6.2 (1–30.7)	1.008 (1.005–1.011)	< 0.001	1.004 (0.999–1.009)	0.149
ESR (mm/h)	46 (21–85.0)	32 (14–58.0)	1.004 (0.995–1.013)	< 0.001	1.002 (0.994–1.011)	0.589
Low-dose corticosteroid use (N [%])	13 (8.3%)	1 (0.05%)	66.458 (7.657–576.811)	< 0.001	76.146 (8.940–648.569)	< 0.001

*IQR* interquartile range, *IGRA* interferon gamma release assays, *LTBI* latent tuberculosis infection, *TB* tuberculosis, *WBC* white blood cell, *CRP* C-reactive protein, *ESR* erythrocyte sedimentation rate.

### Comparison of indeterminate QFT-GIT results by age group

To more accurately analyze the relationship between age and risk of obtaining indeterminate IGRA results, we divided immunocompetent children into three age groups: group A (< 4 years old, n = 744), group B (5 to 9 years old, n = 429), and group C (10 to 18 years old, n = 864).

**Figure 2:**
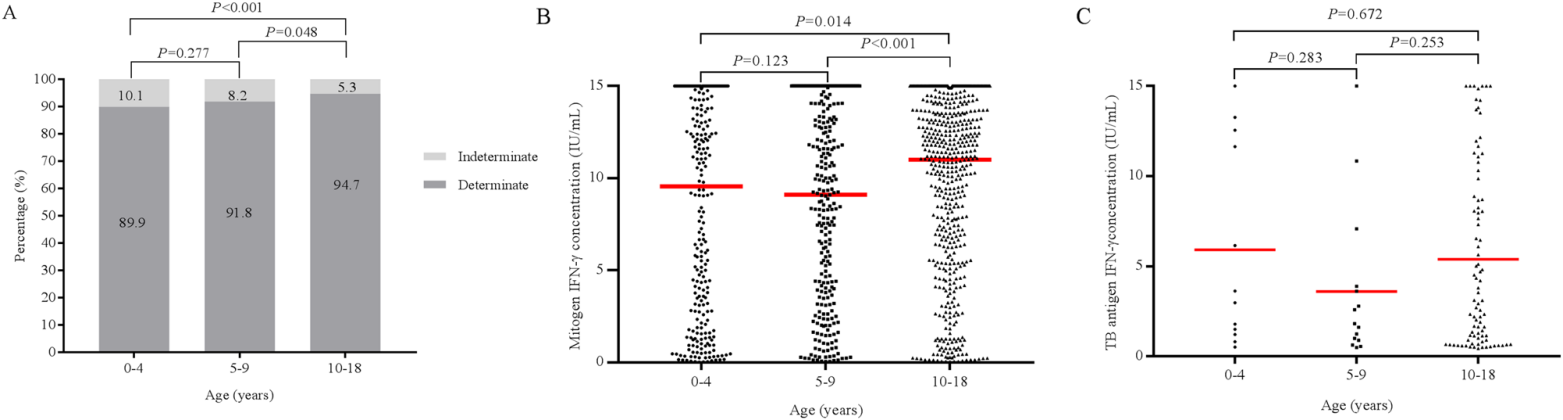
(**A**) Proportion of indeterminate QFT-GIT results in group A (< 4 years old), B (5 to 9 years old), and C (10 to 18 years old). (**B**) IFN-γ response to the mitogen control PHA (censored at 15 IU/mL) according to the age group. Red lines indicate the median values. *P* values were calculated using the Mann–Whitney U test. (**C**) IFN-γ response to the TB antigens (censored at 15 IU/mL) according to age of children with a positive QFT-GIT result. The red lines indicate the median values. *P* values were calculated using the Mann–Whitney U test. *QFT-GIT* QuantiFERON-TB Gold In-Tube, *PHA* phytohemagglutinin, *IFN-γ* interferon-gamma, *TB* tuberculosis.

The proportion of indeterminate results was significantly lower in group C than groups A and B (5.3% vs. 10.1% at *P* < 0.001 and 5.3% vs. 8.2% at *P* = 0.048, respectively) (Fig. 2A). Additionally, the median response to the mitogen control phytohemagglutinin (PHA) (censored at 15 IU/mL) was significantly higher in group C than that in groups A and B (*P* = 0.014 and *P* < 0.001, respectively) (Fig. 2B).

Figure 2C depicts the median IFN-γ response (censored at 15 IU/mL) to the TB antigen among children with a positive QFT-GIT result. The median response to the TB antigen did not differ significantly among the three groups.

### Comparison of indeterminate QFT-GIT results with or without low-dose steroid use

**Figure 3:**
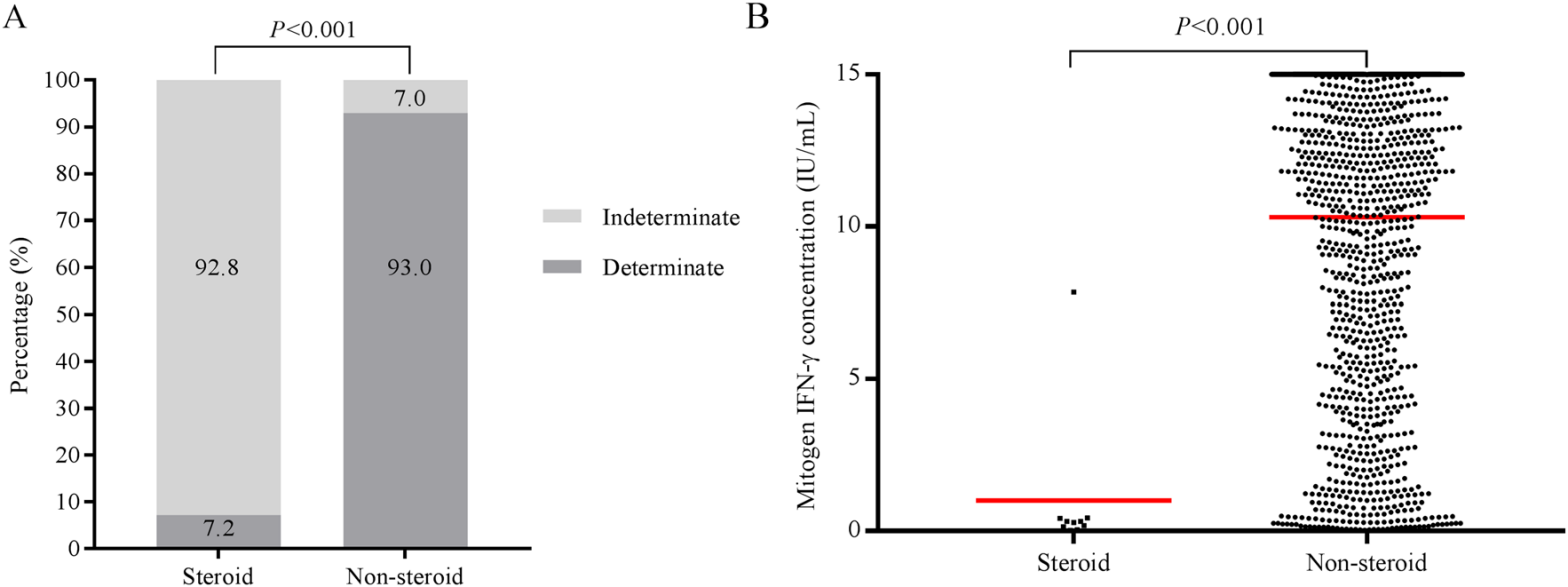
(**A**) Proportion of indeterminate QFT-GIT results in the low-dose steroid group and non-steroid group. (**B**) IFN-γ response to mitogen control PHA (censored at 15 IU/mL) in children with and without low-dose steroid therapy. The red lines indicate the median values. *P* values were calculated using the Mann–Whitney U test. *QFT-GIT* QuantiFERON-TB Gold In-Tube, *IFN-γ* interferon-gamma.

Individuals in the low-dose steroid group had a significantly higher proportion of indeterminate results than those in the non-steroid group (92.8% vs. 7.0%; *P* < 0.001) (Fig. 3A). The median response to the mitogen control PHA (censored at 15 IU/mL) was significantly lower in the low-dose steroid group than that in the non-steroid group (*P* < 0.001) (Fig. 3B). Since there were no positive QFT-GIT results in the steroid group, we did not compare the IFN-γ response to TB antigens in positive samples between the two groups.

## Discussion

This study focused on the risk of indeterminate QFT-GIT assay results in immunocompetent children. Previous studies have reported that an immunocompromised status contributes to indeterminate QFT-GIT results for children^7^. We found that a younger age, high WBC counts, low albumin levels, and low-dose steroid use were significantly associated with indeterminate QFT-GIT results for immunocompetent children.

Even in healthy children, low-dose steroids are prescribed for a variety of anti-inflammatory therapeutic purposes. The effects of these low-dose steroids on IGRA testing in immunocompetent children can be overlooked. Our study showed that using low doses of steroids had the highest OR for the indeterminate results compared to all the other significant risk factors. Supplementary Table S1 online demonstrates that the median dose and duration of steroid use was equivalent to 0.7 mg/kg per day prednisone (IQR 0.7–0.7) and 1 day (IQR 1.0–1.0), respectively. In the present study, most cases treated with low-dose steroids were used as adjuvant treatments for pneumonia (9/14), dyspnea in asthma (3/14), and acute respiratory distress syndrome (1/14). As a common cause of pneumonia, *Mycoplasma pneumoniae* was found in 4 cases, whereas parainfluenza virus was found in 1 case, and *Streptococcus pneumoniae* was found in 1 case. In the remaining three cases, the causative pathogen was not determined. Nevertheless, most IGRA tests were performed to screen TB as a differential diagnosis in these patients with pneumonia that presented with severe symptoms that did not respond to common antibiotics. However, adverse effects of glucocorticoids on the immune system are both dose- and duration-dependent^10–13^. Less than 1 mg/kg per day of prednisone is considered a low-to-moderate dose for children^10^. Although even low-dose steroids may adversely affect the functioning of the immune system in some individuals, the use of the lowest dose of glucocorticoids for the shortest period of time has not been implicated in increasing the risk of severe immunosuppression. Therefore, administering killed or attenuated vaccines can proceed normally in children receiving low doses of steroids^14–17^. Our results suggest that even low doses of steroids can significantly impact QFT-GIT results; therefore, prior to QFT-GIT testing, it is important to check whether patients have been administered steroids.

The QFT-GIT assay is an in vitro blood test based on the release of IFN-γ by T cells in response to stimulation with TB-specific antigens. Thus, IGRA testing in HIV-infected individuals with a reduced T cell count is more likely to result in indeterminate results^18^. Glucocorticoids impair a variety of T cell functions; therefore, high doses can induce T cell apoptosis, which leads to the inhibition of Th1-derived cytokines, such as IFN-γ, interleukin (IL)-2, IL-10, and tumor necrosis factor (TNF)^19^. In two ex vivo models^19, 20^, dexamethasone altered QFT-GIT assay results from positive to negative in 30–40% of the subjects. Several studies in adults have reported that patients receiving at least one immunosuppressive drug, including steroids, were at an increased risk of obtaining indeterminate results^5, 21–24^. However, these studies were conducted in adult patients with underlying diseases, such as inflammatory bowel disease (IBD) or autoimmune disease, which have been commonly treated using steroids. Therefore, it was necessary to interpret the results carefully, as it was possible that the patients’ underlying diseases affected their QFT-GIT results. In addition, long-term steroid use is likely responsible for the immunosuppression observed in the aforementioned studies. However, our study revealed the effect of low-dose steroid use on indeterminate QFT-GIT results; patients with underlying diseases and those receiving immunosuppressive treatments were excluded. Our findings suggest that even low-dose steroids can reduce T cell function and impact the IGRA response; thus, IGRA tests should be performed prior to the use of steroids and in patients that may benefit from its biologics.

The results of the present study correspond with those of earlier studies which reported that young age was independently associated with a higher risk of obtaining indeterminate QFT-GIT results^5, 7, 8^. In particular, we observed that the response to the mitogen control PHA was significantly higher in children older than 10 years compared with that in children younger than 10 years old. These findings are supported by previous studies that have observed an age-associated increase for IFN-γ concentrations in healthy children^25, 26^.

However, there was no difference in the IFN-γ response to TB antigens among the age groups. These findings suggest that a younger age does not affect T-lymphocyte release of IFN-γ in response to a TB-specific antigen. Interestingly, a previous study reported similar results in that TB antigen-induced IFN-γ responses were significantly higher in children than adults and that insufficient IFN-γ production in response to PHA stimulation was more common in children than in adults^5^. Although it is not clear why this discrepancy occurs, these findings suggest that for children, the QFT-GIT assay requires correction for the positive control (Mitogen) according to the age group, but a correction is unnecessary for TB antigens.

Regarding the influence of laboratory findings on the QFT-GIT test, our results demonstrated that high WBC counts and low albumin levels were significantly associated with indeterminate results. However, since the median value for the WBC count falls within the normal range for a child's age in both the indeterminate and determinate groups, and the OR value was not large, the WBC count was considered to have little effect on the IGRA test results. In previous studies, hypoalbuminemia was associated with indeterminate QFT-GIT results in critically ill adults or patients with IBD^27, 28^. Albumin is a well-known negative acute phase reactant because its level decreases with inflammation, in which various relevant cytokines, such as IL-1, IL-6, and TNF-α, suppress the synthesis of albumin^29, 30^. However, since our study was conducted for immunocompetent children with no evidence of chronic inflammation, hypoalbuminemia may have affected the IGRA test results. Therefore, further research is needed to elucidate the mechanisms underlying the effects of low albumin levels on IGRA testing.

This study had several limitations. First, possible biases may have occurred due to the retrospective and single-center nature of this study. We attempted to reduce this effect by including a large cohort of immunocompetent children in the analysis. Second, we could not evaluate factors external to the patient, such as specimen handling and processing. In addition, the age window for children in the determinate and indeterminate groups may be too broad to serve as a real representation of the WBC profile that changes significantly between these different age groups. Third, since the IFN-γ response to the TB antigen by age group was analyzed among children with a positive QFT-GIT result, this resulted in a relatively small group size. Moreover, the IFN-γ concentration was censored when the value exceeded 15 IU/mL; subsequently, analyzing such comparisons were limited, especially as the results were influenced by the censored data. Additional prospective large-scale studies, including assessing factors external to the patients, are required to resolve these limitations. Nevertheless, our study identified factors associated with inconclusive diagnostic test results from conventional TB tests, which highlights the contribution of our results given the importance of accurate and timely diagnosis of TB. However, the findings from this study should be further verified using other diagnostic methodologies to demonstrate that our results are universally applicable across testing systems and not unique to the IGRA-GIT platform.

In conclusion, we identified that 7.7% of immunocompetent children produced indeterminate IGRA results and that factors, including younger age, high WBC count, low albumin levels, and low-dose steroid use, were associated with the attainment of indeterminate QFT-GIT results. Notably, the highest OR was observed for low-dose steroid use. Therefore, our study highlights the importance of screening for steroid use, even in low doses, prior to performing IGRAs for immunocompetent children.

## Materials and methods

### Study population

We retrospectively reviewed the medical charts of 2707 children, aged ≤ 18 years, who underwent QFT-GIT between September 2006 and July 2017 at Severance Hospital, Seoul, South Korea. Electronic health record (EHRs) were searched for all orders of the QFT-GIT assay conducted during the study period. Data extracted from the EHRs included the patient identification (ID) number, demographic variables, such as age and gender at sampling, and QFT-GIT results. Using the patient ID number, their charts were reviewed to indicate the initial reason for conducting the QFT-GIT assay, relevant laboratory data, use of systemic steroids or immunosuppressive agents, and immune status. Immunocompromised patients or individuals with no information regarding immune status were excluded from the present study. Additionally, the following exclusion criteria were used: patients with (1) known primary immune deficiencies; (2) malignancies and receiving chemotherapy or immunosuppressive therapy; (3) hematopoietic stem cell or solid organ transplants; (4) inflammatory disorders and receiving systemic immunosuppressive treatment, including glucocorticoid dose equivalent to prednisone ≥ 1 mg/kg per day or long-term use (2 weeks or more) regardless of dose, as well as azathioprine, methotrexate, and cyclophosphamide; (5) asplenia; (6) HIV; and (7) unknown immune status. Immunocompetent children were defined as patients without the immunocompromised factors as described above. The charts of patients receiving low doses of steroids (< 1 mg/kg per day of prednisone) for less than 2 weeks were carefully reviewed and included in the study, unless they fulfilled one or more exclusion criteria. The demographic and clinical characteristics of children receiving low-dose systemic steroids included in the present study are presented in Supplementary Table S1.

### QFT-GIT assay

QFT-GIT assays were performed and interpreted according to the manufacturer’s instructions in a fully accredited diagnostic laboratory in Severance Hospital. Briefly, whole blood was collected in 3 test tubes [negative control antigen (Nil), positive control antigen (Mitogen), and TB-specific antigens (ESAT-6, CFP-10, and TB7.7)] and incubated at 37 °C for 16–24 h. After centrifugation, IFN-γ production was measured via enzyme-linked immunosorbent assay (ELISA). Data were converted to IU/mL of IFN-γ. Indeterminate results were defined as per manufacturer’s instructions, i.e., failure of the positive control (Mitogen-Nil < 0.5 IU/mL) or negative control (Nil > 8 IU/mL).

### Statistical analysis

The data were analyzed using SAS software, Version 9.4 of the SAS System for Unix. Copyright 2020 SAS INSTITUTE INC. SAS and all other SAS INSTITUTE INC. product or service names are registered trademarks or trademarks of SAS INSTITUTE INC., Cary, NC, USA. Descriptive statistics were used to express continuous variables using median and interquartile range (IQR) and categorical variables using frequency and percentage. The chi-squared test (Fisher’s exact test) and Mann–Whitney U test were performed to compare categorical and continuous variables, respectively. Potential factors associated with the attainment of indeterminate results were identified using univariable logistic regression analysis. Multivariable logistic regression analysis was used to generate ORs and 95% CIs using significant variables (*P* < 0.05) identified in the univariable model and suggested potential risk factors. The Mann–Whitney U test was used to compare median TB antigen/mitogen values between two age groups. Additionally, the Kruskal–Wallis test was used to make comparisons among the three age groups. *P* values less than 0.05 were considered statistically significant.

### Ethics approval and consent to participate

This study was performed in accordance with institutional and national regulations and approved by the institutional review board (IRB) of the Yonsei University Health System (IRB approval number 4-2020-0208) that waived requirements for informed consent due to the retrospective nature of the study.

## Supplementary Information

Supplementary Table S1.

## Data Availability

The datasets used and/or analyzed in the present study are available from the corresponding author upon reasonable request.
