# Supplementary material for: Low-dose steroids are associated with indeterminate QuantiFERON-TB Gold In-Tube assay results in immunocompetent children
Source: Sci Rep. 2021 Mar 19;11:6468. doi: 10.1038/s41598-021-86053-0 (PMC7979822; doi:10.1038/s41598-021-86053-0)
Supplement: Supplementary file 1 — Supplementary Table S1. [file 41598_2021_86053_MOESM1_ESM.docx]

**Supplementary Information**

**Low-dose steroids are associated with indeterminate QuantiFERON-TB Gold In-Tube assay results in immunocompetent children**

**Kyu Ho Kim, Ji-Man Kang, and Jong Gyun Ahn ***

Department of Pediatrics, Severance Children’s Hospital, Yonsei University College of Medicine,

50-1 Yonsei-ro, Seodaemun-gu, Seoul 03722, Korea

* [JGAHN@yuhs.ac](mailto:JGAHN@yuhs.ac)

**Supplementary Table S1.** Clinical characteristics of children receiving low-dose systemic steroids

|  | Age at sampling (years) | Sex | Type of steroid used | Steroid dosage (mg/kg)  (calculated as prednisone equivalent) | Duration of steroid use (d) | Reasons for using steroids | Main diagnosis | Reason for IGRA test | Chest X-ray findings | WBC count (/μL) | CRP  (mg/L) | ESR (mm/hr) | TB meningitis suspicion | Admission to ICU |
| --- | --- | --- | --- | --- | --- | --- | --- | --- | --- | --- | --- | --- | --- | --- |
| Patient 1 | 14 | Female | Prednisone | 0.05 mg | 13 | Use in dermatology for inflammatory skin rash | Enteritis | Unknown | Normal | 6,340 | 7.5 | 4 | No | No |
| Patient 2 | 3 | Female | Dexamethasone | 0.7 mg | 1 | Adjunctive treatment of MRMP | Mycoplasma Pneumonia | Screening test for TB as a differential diagnosis of atypical pneumonia. | Patchy consolidation in both lungs dominant in right lung | 13,660 | 44.6 | 37 | No | No |
| Patient 3 | 12 | Male | Dexamethasone | 0.7 mg | 1 | Improvement of breathing difficulties | Cough variant asthma | Screening test for TB as a differential diagnosis of chronic cough | Normal | 9,500 | 1 | 16 | No | No |
| Patient 4 | 2 | Female | Dexamethasone | 0.7 mg | 1 | Adjunctive treatment of severe pneumonia | PIV Pneumonia | Screening test for TB as a differential diagnosis of atypical pneumonia. | Subtle peribronchial infiltration in both perihilar and RLLF | 6,050 | 4.8 | 42 | No | No |
| Patient 5 | 2 | Female | Dexamethasone | 0.7 mg | 1 | Adjunctive treatment of severe pneumonia | Pneumococcal  Pneumonia | Screening test for TB as a differential diagnosis of atypical pneumonia | Decreased aeration in left lung with total haziness | 10,040 | 375 | 120 | No | No |
| Patient 6 | 4 | Male | Dexamethasone | 0.7 mg | 1 | Adjunctive treatment of MRMP | Mycoplasma  Pneumonia | Screening test for TB as a differential diagnosis of atypical pneumonia | Consolidation/collapse in right upper lung | 11,350 | 16.1 | 40 | No | No |
| Patient 7 | 4 | Male | Dexamethasone | 0.7 mg | 1 | Improvement of breathing difficulties | Asthma | Screening test for TB as a differential diagnosis of hemoptysis | Normal | 5,250 | 2.5 | 18 | No | No |
| Patient 8 | 8 | Male | Dexamethasone | 0.7 mg | 1 | Adjunctive treatment of severe pneumonia | Pneumonia | Screening test for TB as a differential diagnosis of atypical pneumonia | Consolidation and collapse in right lung and right pleural effusion | 8,310 | 117 | ND | No | No |
| Patient 9 | 10 | Male | Dexamethasone | 0.7 mg | 1 | Adjunctive treatment of severe pneumonia | Pneumonia | Screening test for TB as a differential diagnosis of atypical pneumonia | Right pleural effusion, pneumonic consolidation of RUL and RLL | 11,490 | 8 | 89 | No | No |
| Patient 10 | 7 | Female | Dexamethasone | 0.7 mg | 1 | Adjunctive treatment of MRMP | Mycoplasma  Pneumonia | Screening test for TB as a differential diagnosis of atypical pneumonia | Consolidation in RUL with right pleural effusion | 54,40 | 352 | 44 | No | No |
| Patient 11 | 12 | Male | Dexamethasone | 0.7 mg | 1 | Improvement of breathing difficulties | Cough variant asthma, Angioedema | Screening test for TB as a differential diagnosis of chronic cough | Normal | 11,650 | 0.3 | 2 | No | No |
| Patient 12 | 7 | Female | Dexamethasone | 0.7 mg | 1 | Adjunctive treatment of ARDS | ARDS | Screening test for TB as a differential diagnosis of ARDS | Alveolar consolidation in both lung periphery | 4,870 | 10.5 | 14 | No | Yes |
| Patient 13 | 8 | Male | Dexamethasone | 0.7 mg | 1 | Adjunctive treatment of MRMP | Mycoplasma Pneumonia | Screening test for TB as a differential diagnosis of atypical pneumonia | Consolidation/collapse in RLL with right pleural effusion | 8,590 | 1.7 | 33 | No | No |
| Patient 14 | 13 | Female | Dexamethasone | 0.7 mg | 1 | Adjunctive treatment of severe pneumonia | Pneumonia | Screening test for TB as a differential diagnosis of atypical pneumonia | Right pleural effusion, r/o pneumonia, RML and RLL | 28,190 | 139.1 | 85 | No | No |

MRMP, macrolide-refractory *Mycoplasma pneumoniae*; PIV, parainfluenza virus; ARDS, Acute respiratory distress syndrome; IGRA, interferon-gamma release assay; TB, tuberculosis; RLLF, right lower lung field; RUL, right upper lobe; RLL right lower lobe; RML, right middle lobe; WBC, white blood cell; CRP, C-reactive protein; ESR, erythrocyte sedimentation rate; ND, not done.
